# Immunoregulatory Effect of Bifidobacteria Strains in Porcine Intestinal Epithelial Cells through Modulation of Ubiquitin-Editing Enzyme A20 Expression

**DOI:** 10.1371/journal.pone.0059259

**Published:** 2013-03-26

**Authors:** Yohsuke Tomosada, Julio Villena, Kozue Murata, Eriko Chiba, Tomoyuki Shimazu, Hisashi Aso, Noriyuki Iwabuchi, Jin-zhong Xiao, Tadao Saito, Haruki Kitazawa

**Affiliations:** 1 Food and Feed Immunology Group, Graduate School of Agricultural Science, Tohoku University, Sendai, Japan; 2 Laboratory of Clinical and Experimental Biochemistry, Reference Centre for Lactobacilli (CERELA-CONICET), Tucuman, Argentina; 3 Laboratory of Animal Breeding and Genetics, Graduate School of Agricultural Science, Tohoku University, Sendai, Japan; 4 Cell Biology Laboratory, Graduate School of Agricultural Science, Tohoku University, Sendai, Japan; 5 Food Science and Technology Institute, Morinaga Milk Industry Co. Ltd, Zama, Kanagawa, Japan; Ecole Normale Supérieur de Lyon, France

## Abstract

**Background:**

We previously showed that evaluation of anti-inflammatory activities of lactic acid bacteria in porcine intestinal epithelial (PIE) cells is useful for selecting potentially immunobiotic strains.

**Objective:**

The aims of the present study were: i) to select potentially immunomodulatory bifidobacteria that beneficially modulate the Toll-like receptor (TLR)-4-triggered inflammatory response in PIE cells and; ii) to gain insight into the molecular mechanisms involved in the anti-inflammatory effect of immunobiotics by evaluating the role of TLR2 and TLR negative regulators in the modulation of proinflammatory cytokine production and activation of mitogen-activated protein kinase (MAPK) and nuclear factor-κB (NF-κB) pathways in PIE cells.

**Results:**

*Bifidobacteria longum* BB536 and *B. breve* M-16V strains significantly downregulated levels of interleukin (IL)-8, monocyte chemotactic protein (MCP)-1 and IL-6 in PIE cells challenged with heat-killed enterotoxigenic *Escherichia coli*. Moreover, BB536 and M-16V strains attenuated the proinflammatory response by modulating the NF-κB and MAPK pathways. In addition, our findings provide evidence for a key role for the ubiquitin-editing enzyme A20 in the anti-inflammatory effect of immunobiotic bifidobacteria in PIE cells.

**Conclusions:**

We show new data regarding the mechanism involved in the anti-inflammatory effect of immunobiotics. Several strains with immunoregulatory capabilities used a common mechanism to induce tolerance in PIE cells. Immunoregulatory strains interacted with TLR2, upregulated the expression of A20 in PIE cells, and beneficially modulated the subsequent TLR4 activation by reducing the activation of MAPK and NF-κB pathways and the production of proinflammatory cytokines. We also show that the combination of TLR2 activation and A20 induction can be used as biomarkers to screen and select potential immunoregulatory bifidobacteria strains.

## Introduction

The intestinal epithelium is a physical barrier that separates trillions of commensal bacteria in the intestinal lumen from the underlying lamina propria and deeper intestinal layers. Although once considered simply a physical barrier, it is becoming increasingly evident that the epithelium plays a crucial role as a regulator of intestinal immune homeostasis. In response to pathogens, intestinal epithelial cells (IECs) may produce a variety of factors that play a crucial role in both the innate and adaptive immune responses in the gut [1]. However, under steady-state conditions, IECs should create a tolerogenic environment to avoid inflammatory-mediated tissue damage. These two functions of IECs – distinguishing among the diverse elements of the intestinal microbiota and responding to pathogenic microorganisms – are determined by pattern recognition receptors (PRRs) such as Toll-like receptors (TLRs) [2], [3].

TLRs are the most extensively studied innate receptors, and their roles in innate and adaptive mucosal immunity are well documented. TLR signaling in IECs is involved in several important mechanisms that are crucial for maintaining a healthy epithelial barrier such as epithelial cell proliferation, maintenance of tight junctions, expression of antimicrobial factors, and modulation of immune responses [4]. However, inappropriate TLR signaling contributes to loss of intestinal tolerance and results in tissue injury. TLRs have been implicated in several immune-mediated and inflammatory diseases. In fact, disruption of anti-inflammatory and immunosuppressive mechanisms in the gut is believed to lead to inflammatory diseases via hyperresponsiveness to the commensal flora. TLR signaling plays an important role in commensal-dependent colitis in several animal models [4], [5].

In recent years, the study of microbe–IEC interactions has unraveled several molecular mechanisms and cellular pathways, showing that this interaction plays a crucial role in the regulation of several immunological functions in the gut. Moreover, better understanding of the host–microbe interactions in the gut has provided new opportunities for preventing and treating a number of inflammatory disorders such as the use of specific probiotic strains to beneficially modulate the intestinal immune system. Probiotic bacteria that can modulate the immune system (immunobiotics) are demonstrably beneficial for treating a variety of mucosal disorders, including inflammatory diseases [5], [6]. In this sense, we have developed and used several *in vitro* tests based in porcine cells to identify anti-inflammatory, potentially immunobiotic strains and to study the mechanisms by which immunobiotics exert their immunoregulatory effects [7]–[9]. We have shown that a mitogenic assay using porcine Peyer’s patches (PPs) and mesenteric lymphoid node (MLN) immunocompetent cells and evaluation of anti-inflammatory activities of lactic acid bacteria in a porcine intestinal epithelial (PIE) cell line are useful for selecting potential immunobiotic strains [7]. In addition, we have demonstrated that PIE cells can be used to study the mechanisms involved in the anti-inflammatory effect of immunobiotics in IECs [2], [10].

The aims of the present study were: i) to select potentially immunomodulatory bacteria that may beneficially modulate the TLR4-triggered inflammatory response in IECs and; ii) to gain insight into the molecular mechanisms involved in the anti-inflammatory effect of immunobiotics by evaluating the role of TLR2 and TLR negative regulators in the regulation of proinflammatory cytokine production and activation of mitogen-activated protein kinase (MAPK) and nuclear factor κB (NF-κB) pathways in PIE cells.

## Results

### Immunomodulatory Activity of Bacterial Strains in Immune and PIE Cells

**Figure 1:**
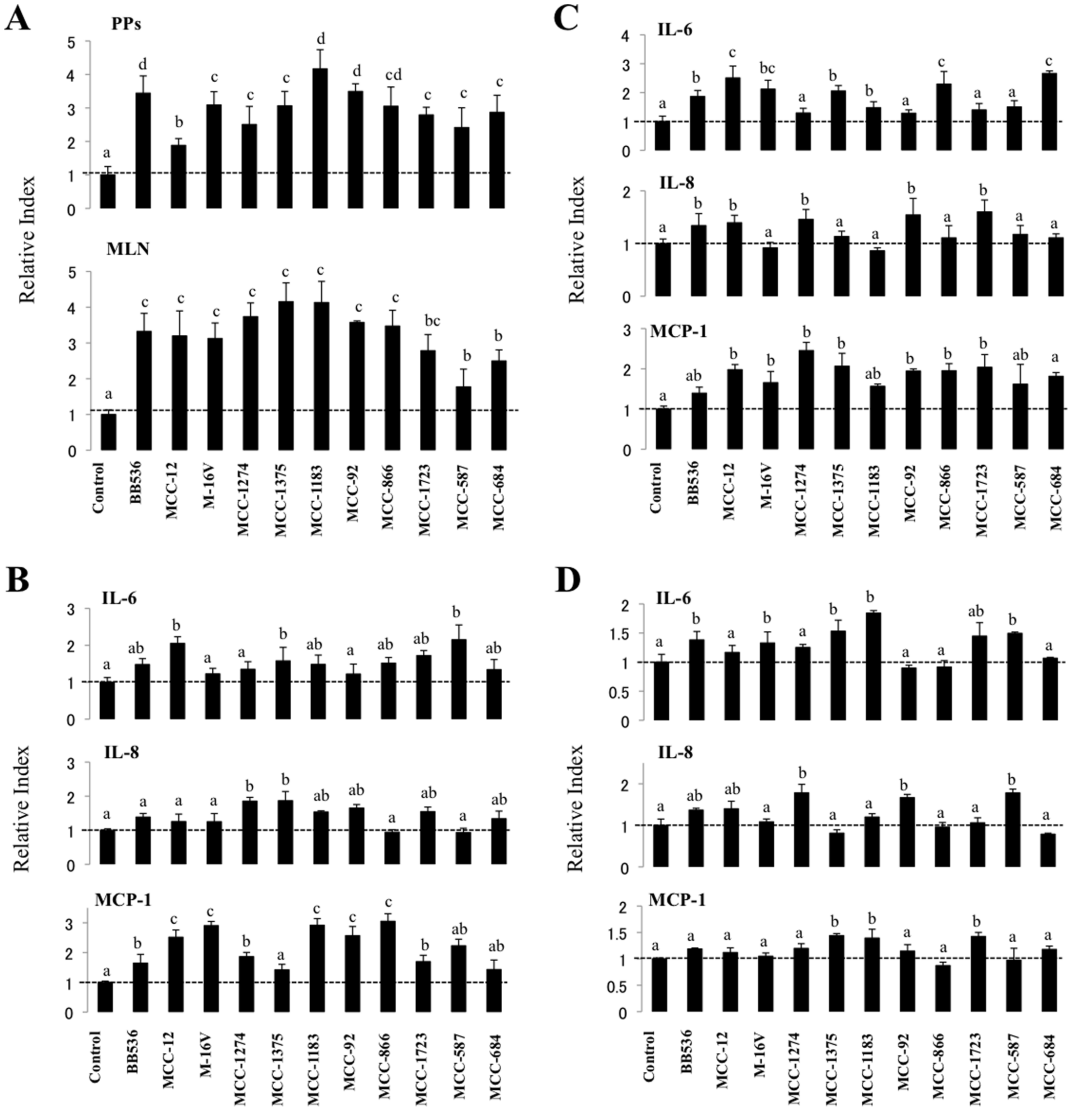
Selection of bacterial strains with immunomodulatory capacities. Bacteria were evaluated by studying (A) the mitogenic assay and expression of cytokines in porcine Peyer_$B!G_(Bs patches (B), mesenteric lymphoid nodes (C), and porcine intestinal epithelial cells (D). Cells were pre-treated with different bacterial strains for 48 h, and then the mitogenic activity or the expression of MCP-1, IL-6, and IL-8 were measured. The results represent four independent experiments. Different letters indicate significant differences (P<0.05).

A commonly used method for studying the immunostimulatory properties of potential probiotic microorganisms is evaluation of the mitogenic activity [7]. We used this method to assess the immunostimulatory capacity of 11 strains of different species of lactobacilli, lactococci, and bifidobacteria using immunocompetent cells isolated from swine PPs or MLNs (Figure 1A). All studied strains significantly increased the mitogenic activity of PPs and MLNs compared to the control group. The effect on PPs was significantly higher for *Bifidobacteria longum* BB536, *Lactobacillus gasseri* MCC-1183, and *B. pseudolongum* MCC-92 compared with the other strains. In addition *L. gasseri* MCC-587 and *L. helveticus* MCC-684 showed the lowest capacity to increase mitogenic activity in MLN cells (Figure 1A).

We also evaluated the capacity of all the strains to modulate the production of interleukin (IL)-6, IL-8, and monocyte chemotactic protein (MCP)-1 in mononuclear cells from PPs (Figure 1B) and MLNs (Figure 1C). IL-6 expression in PP mononuclear cells was upregulated with the MCC-12, MCC-1375, and MCC-587 strains, whereas IL-8 mRNA levels were increased only with MCC-1274 and MCC-1375 treatment. On the contrary, several strains upregulated MCP-1 expression in PP cells, including MCC-12, M-16V, MCC-1183, MCC-92, and MCC-866, which showed the highest capacity (Figure 1B). Seven strains upregulated IL-6 mRNA levels in MLN mononuclear cells, including MCC-12, MCC-866, and MCC-684, which showed the strongest effect (Figure 1C). In addition, BB536, MCC-12, MCC-1274, MCC-92, and MCC-1723 upregulated the expression of IL-8 in MLN cells. All strains increased MCP-1 expression in MLN cells with the exception of BB536 and MCC-1183 (Figure 1C).

We studied the effect of the strains on cytokine production in PIE cells and observed that several strains upregulated the expression of IL-6 including BB536, M-16V, MCC-1375, MCC-1183, and MCC-587 (Figure 1D). IL-8 mRNA levels were increased in PIE cells stimulated with MCC-1274, MCC-92, and MCC-587 strains, whereas MCC-1375 and MCC-1183 were the only treatments that upregulated MCP-1 expression (Figure 1D).

### Identification of Bacterial Strains that Modulate the Proinflammatory Response in PIE Cells

**Figure 2:**
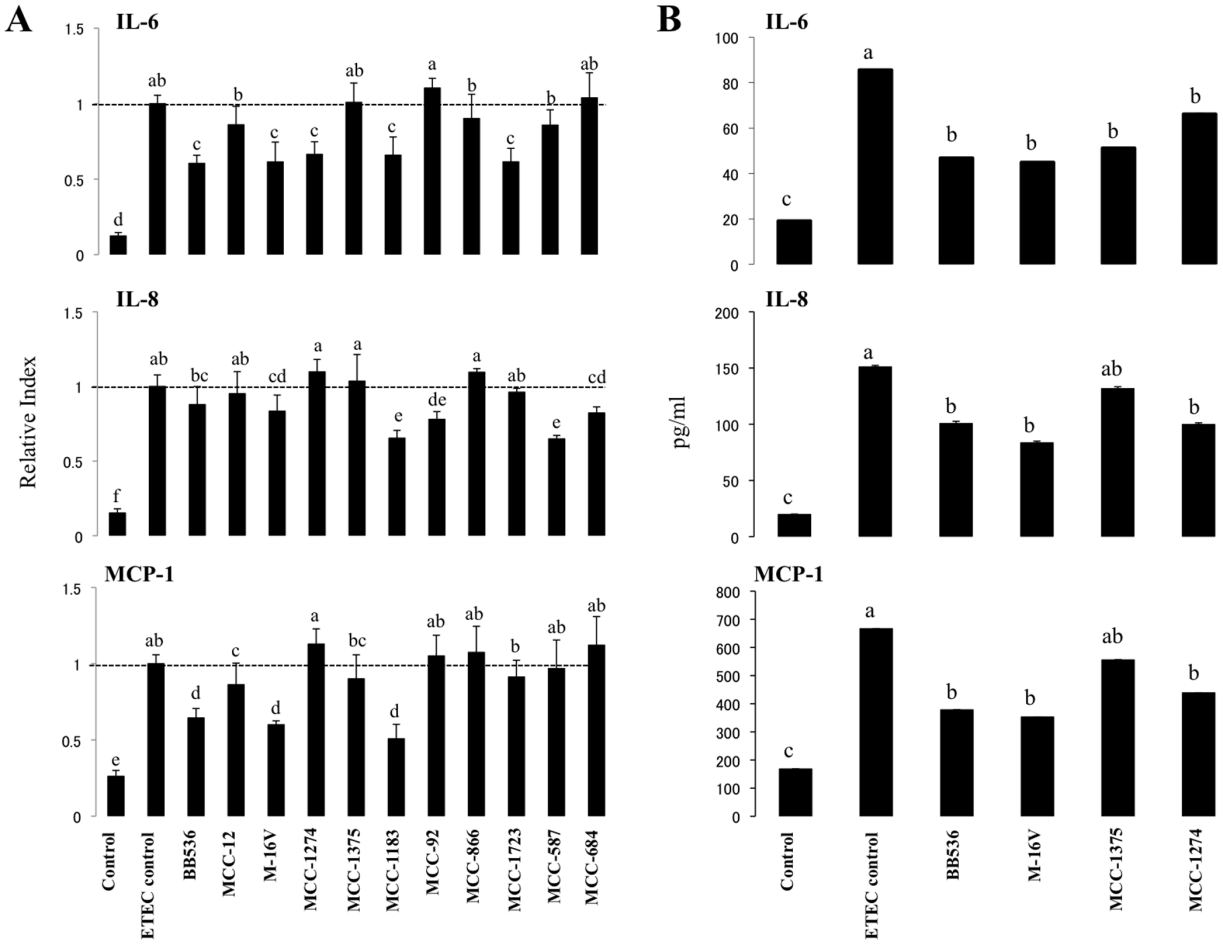
Expression of cytokines in porcine intestinal epithelial (PIE) cells after stimulation with heat-stable Enterotoxigenic *Escherichia coli* (ETEC) pathogen-associated molecular patterns (PAMPs). PIE cells were pre-treated with different bacterial strains for 48 h and stimulated with heat-stable ETEC PAMPs. The expression of MCP-1, IL-6, and IL-8 mRNAs (A) and proteins levels (B) were studied at 12 h post-stimulation. The results represent four independent experiments. Different letters indicate significant differences (P<0.05).

We next evaluated the potential anti-inflammatory effect of bacterial strains in PIE cells with the aim of finding the strain with the highest immunomodulatory capacity in this system. Because TLR4 is expressed at a higher level than other TLRs in PIE cells [8], we used heat-stable enterotoxigenic *Escherichia coli* (ETEC) pathogen-associated molecular patterns (PAMPs) as the inflammatory challenge. The ETEC 987P strain used in this study does not express ﬂagellin, and we have demonstrated that the main molecule responsible for the inflammatory response triggered by this bacterium is the lipopolysaccharide (LPS) present on its surface [2], [9]. PIE cells were cultured for 3 days and then challenged with heat-stable ETEC PAMPs. In addition, individual PIE cell cultures were stimulated with a single bacterial strain for 48 h and then challenged with ETEC PAMPs. Twelve hours after stimulation, we determined mRNA levels of several cytokines (Figure 2A). As we described previously [2], stimulation of PIE cells with heat-stable ETEC PAMPs significantly increased the mRNA levels of the proinflammatory cytokines MCP-1, IL-6, and IL-8 (Figure 2A). Strains BB536, M-16V, MCC-1274, MCC-1183, and MCC-1723 significantly downregulated expression of IL-6 in response to heat-stable ETEC PAMPs challenge (Figure 2A). Both *L. gasseri* MCC-1183 and MCC-587 strains had the highest capacity to downregulate IL-8, whereas BB536, M-16V, and MCC-1183 strongly downregulated MCP-1. We selected *B. longum* BB536 and *B. breve* M-16V for further experiments to test their immunoregulatory effects on PIE cells. We also selected *B. breve* MCC-1274 (moderate anti-inflammatory effect) and *L. paracasei* MCC-1375 (negative control).

To further confirm the immunoregulatory effect of *B. longum* BB536 and *B. breve* M-16V and to detect protein to support the transcriptional data for the selected cytokines, we conducted enzyme-linked immunosorbent assays (ELISAs) to evaluate the levels of IL-6, IL-8, and MCP-1 proteins (Figure 2B). PIE cells were again stimulated with *B. longum* BB536, *B. breve* M-16V, *B. breve* MCC-1274, or *L. paracasei* MCC-1375. Forty-eight hours later, cells were challenged with heat-stable ETEC PAMPs, which significantly increased levels of IL-6, IL-8, and MCP-1 proteins in supernatants of bacteria-pretreated and control PIE cells. However, pretreatment of PIE cells with *B. longum* BB536, *B. breve* M-16V, or *B. breve* MCC-1274 significantly reduced the protein levels of IL-6, IL-8, and MCP-1 (Figure 2B). Treatment of PIE cells with *L. paracasei* MCC-1375 reduced the production IL-6 in response to ETEC PAMPs challenge, but it did not modify IL-8 and MCP-1 levels compared to ETEC control PIE cells (Figure 2B).

### Effect of Bacterial Strains on MAPK and NF-κB Pathways in PIE Cells

**Figure 3:**
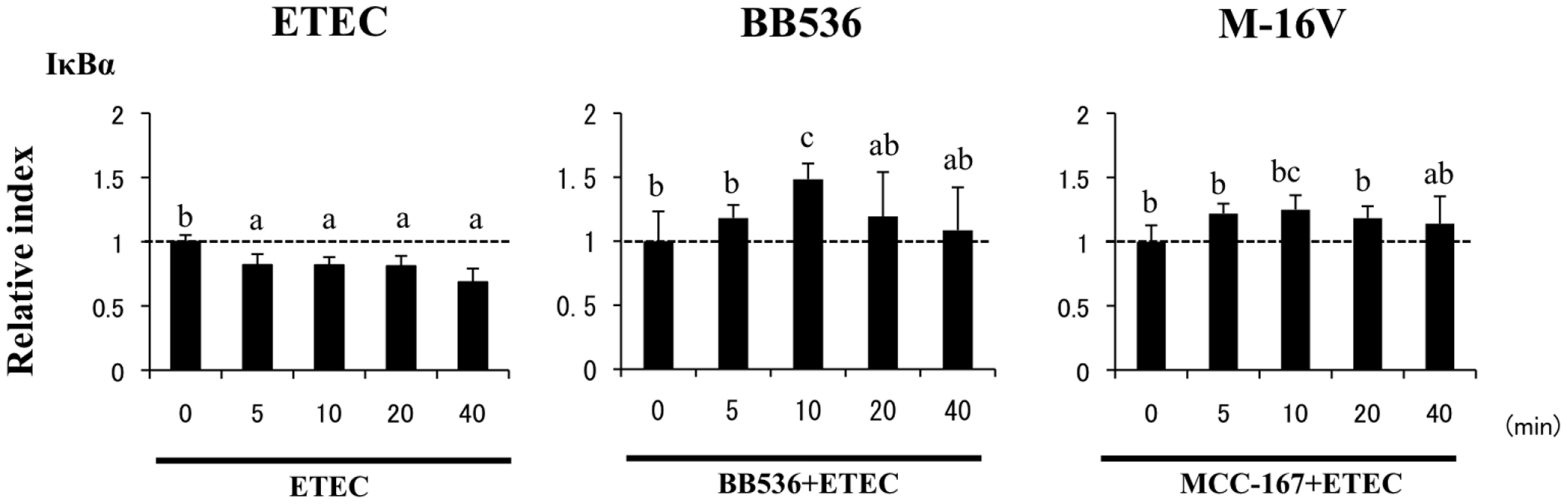
Western blot analysis of NF-κB pathway activation on porcine intestinal epithelial (PIE) cells after challenge with heat-stable Enterotoxigenic *Escherichia coli* (ETEC) pathogen-associated molecular patterns (PAMPs). PIE cells were pre-treated with different bacterial strains for 48 h and stimulated with heat-stable ETEC PAMPs. Levels of the counter-regulatory factor IκBα were studied at the indicated times post-stimulation. Different letters indicate significant differences (P<0.05).

We next evaluated whether *B. longum* BB536 and *B. breve* M-16V attenuated the heat-stable ETEC PAMP-mediated proinflammatory response by modulating the NF-κB pathway (Figure 3). Challenge of PIE cells with heat-stable ETEC PAMPs significantly reduced the levels of the counter-regulatory factor IκBα after 5 to 40 min, indicating activation of the NF-κB pathway. PIE cells previously stimulated with *B. longum* BB536 showed significantly higher levels of IκBα after 10 min, and did not show significant degradation of IκBα after 20 or 40 min, indicating an inhibitory effect of the NF-κB pathway (Figure 3). In addition, no significant modifications of IκBα were observed in *B. breve* M-16V-treated PIE cells.

**Figure 4:**
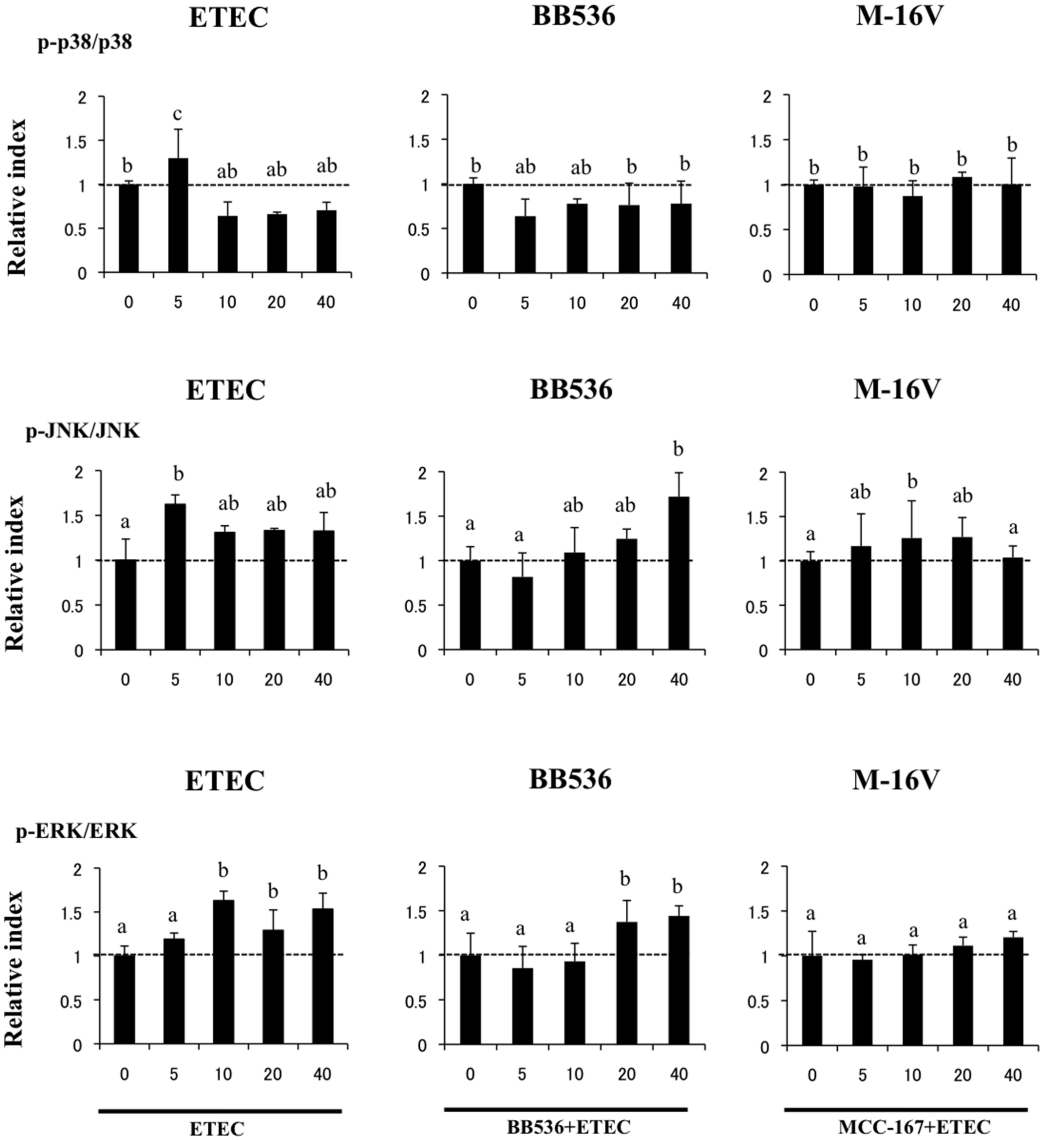
Western blot analysis of activation of p38 and JNK and ERK mitogen-activated protein kinases in porcine intestinal epithelial (PIE) cells after challenge with heat-stable Enterotoxigenic *Escherichia coli* (ETEC) pathogen-associated molecular patterns (PAMPs). PIE cells were pre-treated with different bacterial strains for 48 h and then stimulated with heat-stable ETEC PAMPs. Phosphorylation of p38, JNK, and ERK was studied at the indicated times post-stimulation. Different letters indicate significant differences (P<0.05).

We also examined the relationship between MAPK activation and the regulation of proinflammatory cytokines in PIE cells by bifidobacteria (Figure 4). PIE cells were stimulated with *B. longum* BB536, *B. breve* M-16V, or control medium, and the activation profiles of p38, extracellular signal-regulated kinase (ERK), and Jun N-terminal protein kinase (JNK) after ETEC challenge were compared. Heat-stable ETEC PAMPs induced the phosphorylation of p38 and JNK after 5 min and the phosphorylation of ERK between 10 and 40 min. *B. longum* BB536- and *B. breve* M-16V-treated PIE cells showed significantly lower levels of phosphorylated p38 after 5 min than control cells (Figure 4). In addition, the time course of JNK phosphorylation induced by heat-stable ETEC PAMPs in PIE cells treated with bifidobacteria was different from that in the control. Increased levels of phosphorylated JNK were observed after 40 and 10 min for *B. longum* BB536- and *B. breve* M-16V-treated PIE cells, respectively (Figure 4). We also observed the phosphorylation of ERK between 20 and 40 min for *B. longum* BB536-treated PIE cells, but no modification of ERK was observed in *B. breve* M-16V-treated PIE cells (Figure 4).

### Effect of Bacterial Strains on Negative Regulators of the TLR Signaling Pathway in PIE Cells

**Figure 5:**
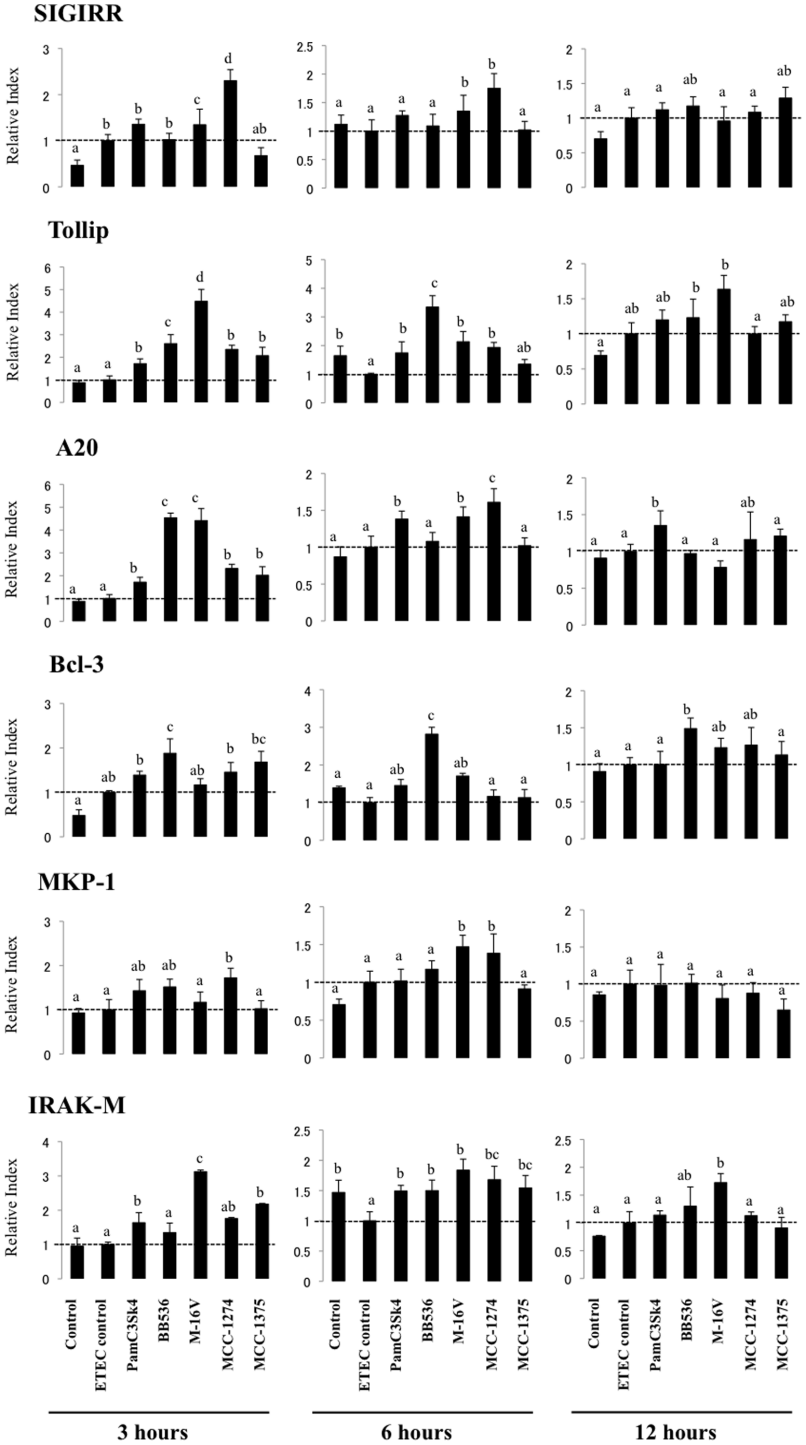
Expression of toll-like receptor negative regulators in porcine intestinal epithelial (PIE) cells. PIE cells were pre-treated with different bacterial strains for 48 h, stimulated with heat-stable ETEC PAMPs, and the expression of IRAK-M, SIGIRR, Bcl-3, MKP-1, Tollip, and A20 negative regulators was studied at the indicated time points. The results represent four independent experiments. Different letters indicate significant differences (P<0.05).

We studied negative regulators that are known to mediate the TLR signaling pathway. PIE cells were stimulated for 48 h with bifidobacteria or *L. paracasei* MCC-1375, and the expression of single immunoglobulin IL-1-related receptor (SIGIRR), Toll interacting protein (Tollip), A20, B-cell lymphoma 3-encoded protein (Bcl-3), mitogen-activated protein kinase 1 (MKP-1), and interleukin-1 receptor-associated kinase M (IRAK-M) was determined with real-time PCR 3, 6, and 12 h after ETEC PAMPs challenge (Figure 5). No significant modifications in the expression of TLR negative regulators after heat-stable ETEC PAMPs challenge were observed compared to basal levels, with the exception of the downregulation of Tollip and IRAK-M 6 h post-challenge (Figure 5). We also observed that M-16V and MCC-1274 had the strongest effect on SIGIRR expression after ETEC PAMPs challenge. These strains significantly increased SIGIRR mRNA levels 3 and 6 h post-challenge (Figure 5). Tollip was significantly upregulated in PIE cells treated with BB536 and M-16V strains at all time points after ETEC PAMPs challenge (Figure 5). In addition, expression of A20 was early and strongly upregulated in PIE cells treated with *B. longum* BB536 and *B. breve* M-16V. A20 mRNA levels were 4-fold higher in bifidobacteria-treated PIE cells than in ETEC controls 3 h post-challenge. *B. breve* MCC-1274 also upregulated A20, but its effect was significantly lower than BB536 and M-16V strains (Figure 5). *B. longum* BB536 was the only strain that significantly upregulated Bcl-3 expression 3, 6, and 12 h post-challenge (Figure 5). In addition, only the MCC-1274 strain upregulated MKP-1 expression 3 and 6 h after heat-stable ETEC PAMPs challenge (Figure 5). M-16V and MCC-1375 increased IRAK-M 3 h after challenge, but the effect of M-16V was significantly higher than that of MCC-1375. Moreover, M-16V was the only strain that significantly upregulated IRAK-M at all time points studied (Figure 5).

We also evaluated the effect of the TLR2 synthetic agonist tripalmitoylated lipopeptide Pam3CysSerLys4 (Pam3CSK4) on the expression of TLR negative regulators in PIE cells after heat-stable ETEC PAMPs challenge. We observed increased levels of A20, Tollip, IRAK-M, and SIGIRR mRNA levels 3 h post-challenge in Pam3CSK4-treated PIE cells (Figure 5).

### Role of TLR2 in the Anti-inflammatory Effect of Bacterial Strains

**Figure 6:**
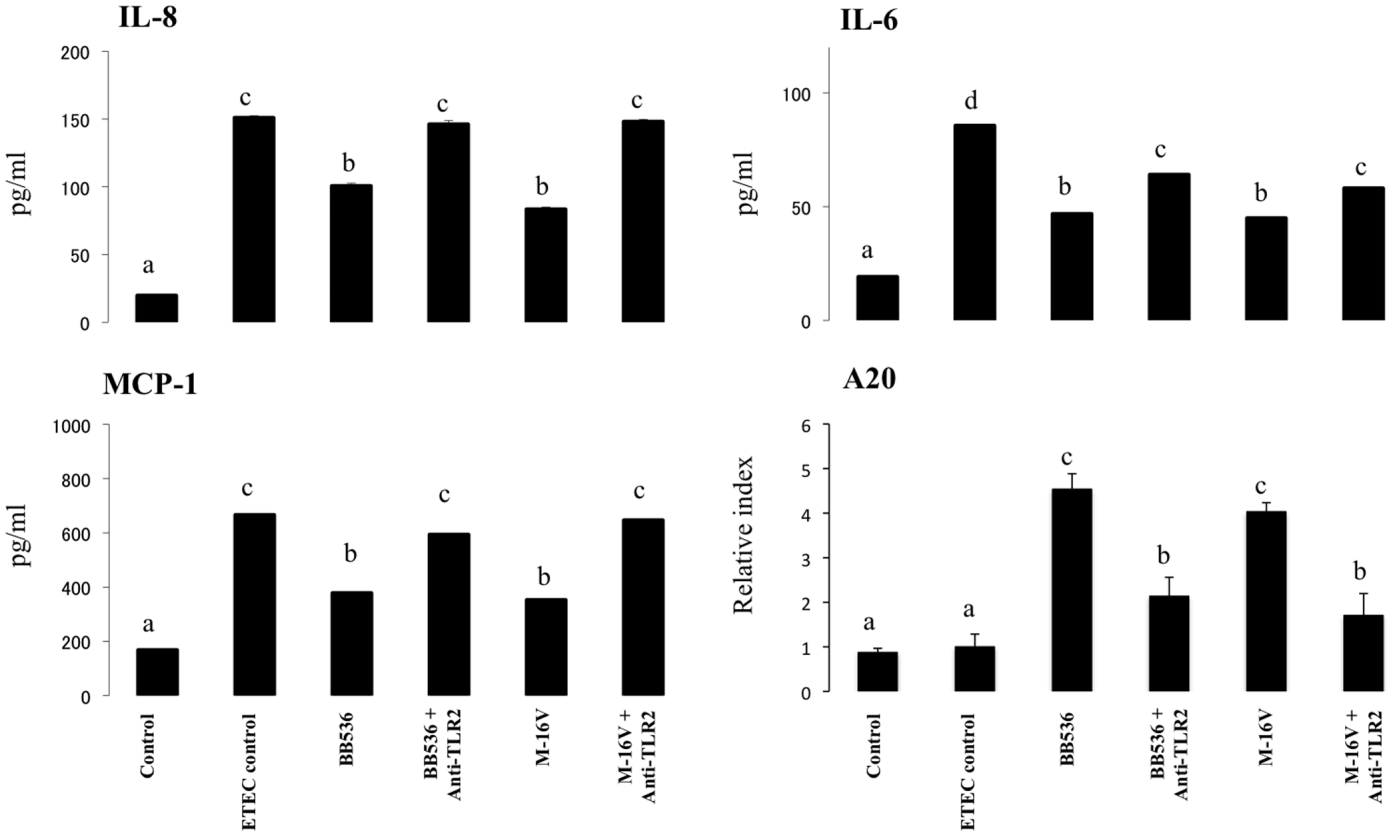
Role of TLR2 in the immunomodulatory effect of *Bifidobacterium longum* BB536 and *Bifidobacterium breve* M-16V in porcine intestinal epithelial (PIE) cells. PIE cells were pre-treated with BB536 or M-16V strains in the presence or absence of anti-TLR2 antibodies. Untreated PIE cells were used as controls. PIE cells were stimulated with heat-stable Enterotoxigenic *Escherichia coli* (ETEC) pathogen-associated molecular patterns (PAMPs), and then the expression of A20 mRNA levels and MCP-1, IL-6, and IL-8 protein levels were studied at 12 h post-stimulation. The results represent four independent experiments. Different letters indicate significant differences (P<0.05).

Several studies have demonstrated that TLR2 is required for some probiotics to exert their immunomodulatory effects [2], [5], [7]. Recently, we demonstrated that stimulation of PIE cells with *Lactobacillus jensenii* TL2937 downregulates the levels of IL-6, IL-8, and MCP-1 in response to heat-stable ETEC PAMPs or LPS challenges and that TLR2 is partially involved in this immunoregulatory effect [2]. Thus, we next evaluated if TLR2 was also involved in the anti-inflammatory effect of *B. longum* BB536 and *B. breve* M-16V. We performed comparative studies with anti-TLR2 blocking antibodies (Figure 6) and observed that anti-TLR2 antibodies abolished the reduction in IL-6, IL-8, and MCP-1 induced by *B. longum* BB536 and *B. breve* M-16V. Considering the early and strong upregulation of A20 that is induced by both *B. longum* BB536 and *B. breve* M-16V in PIE cells and our previous studies demonstrating that the induction of this TLR negative regulator depends on TLR2 activation [2], we also performed blocking experiments to evaluate the role of both TLR2 and A20 in the immunoregulatory effect of bifidobacteria. As shown in Figure 6, the use of anti-TLR2 antibodies abolished the upregulation of A20 induced by *B. longum* BB536 and *B. breve* M-16V.

## Discussion

Growing evidence suggests that some probiotics have immunomodulatory properties and that these properties are strain dependent. In addition, studies comparing the immunoregulatory effect of bifidobacteria and lactobacilli have shown in general that bifidobacteria are better microorganisms for inducing tolerance and exerting anti-inflammatory activities. Our current results are consistent with these general considerations of probiotics. Of the strains evaluated here, two bifidobacteria strains strongly regulated the inflammatory response triggered by TLR4 activation in PIE cells, whereas of the group of lactobacilli and lactococci, only *L. gasseri* MCC-1183 achieved this effect. In addition, we observed that *B. breve* M-16V and *L. gasseri* MCC-1183 showed higher immunoregulatory activity in PIE cells compared with *B. breve* MCC-1274 and *L. gasseri* MCC-587, respectively, confirming that the immunoregulatory activity of probiotics is strain dependent.

Recent studies have shown that *B. infantis* 52486 and *B. longum* SP07/3 strains increase the IL-10/IL-12 ratio in peripheral blood mononuclear cells, whereas the known probiotic strains *L. casei* Shirota and *L. rhamnosus* GG strains decrease this ratio [11]. Probiotic lactobacilli strains have thus been suggested to promote T helper 1 cytokines, whereas bifidobacterial strains tend to produce a more anti-inflammatory profile [12]. Lactobacillus strains are generally considered to induce proinflammatory cytokines, such as IL-12 and interferon-γ, as well as anti-inflammatory cytokines, such as IL-10, whereas *Bifidobacterium* strains usually induce IL-10 better than lactobacilli [7], [13], [14]. In the present study, we selected bifidobacteria strains to gain insight into the mechanism involved in the immunoregulatory effect of immunobiotics in PIE cells.

**Figure 7:**
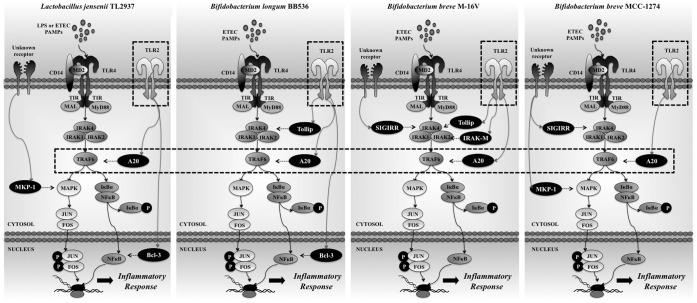
Proposed mechanism for the immunomodulatory effect of *Bifidobacterium longum* BB536 and *Bifidobacterium breve* M-16V in porcine intestinal epithelial cells. The effect of bifidobacteria strains is compared with the previously studied immunobiotic strain, *Lactobacillus jensenii* TL2937 [2].

We demonstrated that *B. longum* BB536 and *B. breve* M-16V significantly downregulated levels of IL-8, MCP-1, and IL-6 in PIE cells challenged with heat-stable ETEC PAMPs. Moreover, we showed that both BB536 and M-16V strains attenuated the proinflammatory response by modulating the NF-κB and MAPK pathways (Figure 7). These modulatory properties of bifidobacteria were also strain dependent. We observed that the BB536 strain had a stronger effect than M-16V on the NF-κB pathway, whereas this last strain was the only one that inhibited activation of the ERK MAPK pathway in PIE cells.

The intestinal immune system must constantly maintain a balance between activation and inhibition of TLRs to avoid detrimental and inappropriate inflammatory responses [4], [5]. Various negative regulatory mechanisms have evolved to attenuate TLR signaling to maintain this balance. In this sense, a growing list of inhibitors of TLR signaling in IECs, including IRAK-M, Tollip, SIGIRR, A20, and peroxisome proliferator-activated receptor-γ (PPARγ), ensure that chronic inflammatory and potentially destructive TLR responses to microbe-associated molecular patterns (MAMPs) from commensal bacteria do not occur [15]. In fact, commensal bacteria can modulate TLR negative regulators in IECs. An anti-inflammatory mechanism has been reported in which activation by commensal *B. thetaiotaomicron* attenuates proinflammatory cytokine expression in IECs by promoting nuclear export of the NF-κB subunit RelA, through a PPARγ-dependent pathway [16]. IECs deficient in SIGIRR are more susceptible to commensal-dependent intestinal inflammation, indicating that the intrinsic expression of SIGIRR by IECs regulates the communication between commensal bacteria and the host immune system [17]. In addition, we have demonstrated that TLR negative regulators play an important role in the anti-inflammatory effect of some immunobiotics strains. We previously showed that *L. jensenii* TL2937 attenuates the expression of proinflammatory cytokines and chemokines caused by LPS challenge in PIE cells by downregulating TLR4-dependent NF-κB and MAPK activation via upregulation of A20, Bcl-3, and MKP-1 [2]. We also reported that sustained upregulation of A20, Tollip, and SIGIRR plays an important role in the anti-inflammatory activity of *L. casei* MEP221114 in poly(I:C)-challenged PIE cells [18]. Therefore, in this work, we evaluated the effect of selected bifidobacteria strains on the expression of several TLR negative regulators. Our findings provide evidence for a key role for the ubiquitin-editing enzyme A20 in the anti-inflammatory effect of immunobiotic bifidobacteria in porcine IECs (Figure 7).

A20 is a zinc finger protein that inhibits activation of NF-κB via inflammatory cytokine receptors [18], [19], TLR [20], [21], and the nucleotide-binding oligomerization domain-containing receptor NOD2 [22]. A20 functions via its two ubiquitin-editing activities, an N-terminal deubiquitinase that removes K63-linked polyubiquitin chains and a C-terminal ubiquitin ligase that facilitates target protein degradation via attachment of K48-linked polyubiquitin chains [19], [23]. These two activities cooperatively downregulate the tumor necrosis factor (TNF)-α receptor-associated factor 6 [24]. Therefore, A20 plays an essential role in the termination of NF-κB signaling in response to TNF-α and microbial products such as LPS [25]. A20 deficiency in enterocytes renders mice sensitive to TNF-α-induced lethal inflammation, leading to disruption of the epithelial barrier and infiltration of commensal bacteria that initiate a systemic inflammatory response [26]. These data suggest that A20 is important for the inhibition of innate immune responses in the gut [15]. In addition, gut decontamination with a mixture of antibiotics with limited oral bioavailability in drinking water markedly reduces A20 protein and mRNA levels in the ileal epithelium of mice [27]. Moreover, partial rather than complete abrogation of A20 expression is likely due to incomplete elimination of intestinal bacteria by the antibiotic treatment [27]. These results show that A20 expression in the epithelium positively correlates with the bacterial load in the lumen. The observations that A20-deficient mice develop severe gut inflammation early in life [28] and that this inflammatory state can be alleviated by antibiotics or knockout of the TLR signaling mediator myeloid differentiation factor MyD88 [29] further support a key role for A20 in intestinal tolerance to the intestinal microbiota.

In our work, the bifidobacteria with the highest capacity to downregulate the expression of inflammatory cytokines in response to heat-stable ETEC PAMPs upregulated A20 in PIE cells. In fact, the most effective strains evaluated in our laboratory, *L. jensenii* TL2937 [2] and bifidobacteria strains BB536 and M-16V, strongly upregulated the ubiquitin-editing enzyme A20 (Figure 7). This finding is of interest because it not only shows a common mechanism for the anti-inflammatory activity of immunobiotics but also provides a potential biomarker for the screening and selection of new immunoregulatory strains.

Our results also show that other negative regulators of inflammatory signaling are redundant with A20 in the activity of immunobiotics. However, the combination of TLR negative regulators that was upregulated in PIE cells was specific for each strain (Figure 7), which would explain the different capacity of BB536 and M-16V strains to modulate the NF-κB and MAPK pathways. This differential effect could certainly be due to the molecular complexity of the different bifidobacteria MAMPs that may contact and activate the various PRRs expressed in PIE cells, resulting in changes in PIE cells that are characteristic for each strain.

We were also interested in evaluating the role of TLR2 in the immunoregulatory effect of bifidobacteria. TLR2 plays an important regulatory role in the recognition of immunoinhibitory bifidobacteria. The supernatant of *B. breve* C50 induces dendritic cell (DC) maturation and prolongs survival through TLR2, with high IL-10 production [30]. Bifidobacteria inhibit the production of IL-6 and TNF-α induced by immunostimulatory lactobacilli in blood immune cells via interaction with TLR2 [31]. In addition, bifidobacteria induce much higher levels of IL-12 and lower IL-10 levels in bone marrow-derived DCs from TLR2^−/−^ mice compared with wild-type DCs [31]. Our results also suggest that TLR2 plays an important role in the anti-inflammatory activity of bifidobacteria because the upregulation of A20 expression and the reduction of proinflammatory mediators in heat-stable ETEC PAMPs-challenged PIE cells were abolished when anti-TLR2 blocking antibodies were used. Moreover, the synthetic agonist for TLR2, Pam3CSK4, significantly increased the levels of A20 in PIE cells. TLR2 can induce tolerance to LPS that is referred to as heterotolerance or cross-tolerance [32]. Therefore, bifidobacteria may induce cross-tolerance in IECs through their interaction with TLR2 and upregulation of A20. In fact, a common mechanism for immunobiotics may be induction of tolerance in PIE cells because we also demonstrated that *L. jensenii* TL2937 regulates the inflammatory response in PIE cells via upregulation of A20 in a TLR2-dependent manner [2].

Using Pam3CSK4, we also demonstrated here that TLR2 activation upregulated the expression of Tollip, Bcl-3, and IRAK-M in PIE cells. We showed that the bifidobacteria strains with the highest capacity to regulate the inflammatory response in PIE cells also upregulated these TLR negative regulators (Figure 7). Therefore, bifidobacteria strains with the capacity to activate TLR2 have a higher ability to induce tolerance in the gut. This idea is supported by our previous work demonstrating that bifidobacteria strains with high capacity to activate TLR2-overexpressing cells also efficiently stimulate IL-10-producing CD4^+^ CD25^high^ Foxp3^+^ Treg cells from swine PPs [7]. Moreover, in this study, we also evaluated the expression of TLR negative regulators in PIE cells treated with *B. breve* MCC-1274, which has a moderate anti-inflammatory effect compared with BB536 and M-16V strains. We observed that upregulation of A20 in *B. breve* MCC-1274-treated PIE cells was lower than in PIE cells pre-stimulated with BB536 or M-16V strains. In addition, *B. breve* MCC-1274 upregulated SIGIRR and MKP-1, TLR negative regulators that seem to not be induced by TLR2 activation but by other unknown receptor(s). These observations further support a key role for TLR2 in the induction of tolerance by immunobiotics in PIE cells.

### Conclusions

The quest for a better understanding of how immunobiotics work has boosted an enormous interest in the molecular processes underlying host-microbe interactions. As recently reviewed [5], [6], the final conclusion of studies that have examined the molecular mechanism of probiotic immunomodulatory activities is that their effect depends on the combination of distinct MAMPs that interact with various PRRs and the associated co-receptors that fine-tune signaling, as well as on the quantity and quality of these MAMPs. Therefore, host–immunobiotics interactions are not univocal but involve the complex interactions of various microbial molecules with various host receptors and adaptor molecules [33]. In this paper, we provide new information regarding the mechanism involved in the anti-inflammatory effect of immunobiotics by demonstrating that several immunoregulatory strains use a common mechanism to induce tolerance in PIE cells. Immunoregulatory strains interact with TLR2, upregulate the expression of A20 in PIE cells, and beneficially modulate the subsequent TLR4 activation by reducing the activation of MAPK and NF-κB pathways and the production of proinflammatory cytokines. Moreover, the results of this work demonstrated that the combination of TLR2 activation and A20 induction can function as biomarkers to screen and select potential immunoregulatory bifidobacteria strains.

## Materials and Methods

### Microorganisms

Bifidobacteria, lactobacilli, and lactococci were provided by Morinaga Milk Industry Co. Ltd (Zama, Japan). Eleven different strains were used in the experiments: *Bifidobacterium longum* BB536; *Bifidobacterium breve* M-16V and MCC-1274; *Bifidobacterium infantis* MCC-12; *Bifidobacterium pseudolongum* MCC-92; *Lactobacillus paracasei* MCC-1375; *Lactobacillus gasseri* MCC-1183 and MCC-587; *Lactococcus lactis* MCC-866 and MCC-1723; and *Lactobacillus helveticus* MCC-648. Bifidobacteria were grown in Man-Rogosa-Sharpe (MRS) broth and agar (Difco, Detroit, MI) supplemented with 0.05% (w/v) cysteine (Sigma, Tokyo, Japan) and incubated at 37°C for 16 h under anaerobic conditions (AnaeroGen; Oxoid, Basingstoke, UK). Cultures were then centrifuged at 1900×*g* for 10 min, and bifidobacteria were washed with phosphate-buffered saline (PBS) and resuspended in Dulbecco’s modified Eagle’s medium (DMEM) at the appropriate concentrations [7]. Lactobacillus strains were grown in MRS medium for 16 h at 37°C, washed with PBS, and resuspended in DMEM at the appropriate concentrations. Lactococcus strains were grown in M17 broth (Difco) supplemented with 0.5% (w/v) lactose (Wako, Osaka, Japan) for 16 h at 37°C, washed with PBS, and resuspended in DMEM at the appropriate concentrations.

ETEC strain 987P was kindly provided by Dr. M. Nakazawa, National Institute of Animal Health (Tsukuba, Japan). ETEC cells were plated into tryptic soy agar (Becton, Dickinson and Company) supplemented with 5% sheep blood (Nippon Biotest Laboratories Inc., Tokyo, Japan). After overnight incubation at 37°C, a single colony was transferred to tryptic soy broth and grown for 24 h at 37°C with shaking (200 rpm). The subcultures of bacteria that had grown to the mid-log phase were centrifuged at 1900×*g* for 10 min at 4°C, washed with PBS, heat-treated at 65°C for 30 min, and resuspended in DMEM as described [2]. We refer to this preparation as heat-stable ETEC PAMPs.

### PIE Cells

PIE cells, which are nontransformed intestinal cultured cells originally derived from intestinal epithelia from an unsuckled neonatal swine [8], were maintained in DMEM (Invitrogen Corporation, Carlsbad, CA) supplemented with 10% fetal calf serum (FCS), 100 mg/ml penicillin, and 100 U/ml streptomycin at 37°C in an atmosphere of 5% CO_2_. PIE cells grow rapidly and are well adapted to culture conditions even without transformation or immortalization. However, the proliferative ability of PIE cells diminishes after 50 passages in culture. Therefore, we used PIE cells only between the 20^th^ and 40^th^ passages in these experiments.

### Isolation of Immunocompetent Cells from Swine PPs and MLNs

Suspensions of porcine PPs or MLN immunocompetent cells were prepared from adult swine intestine as described [7], [9]. This study was carried out in strict accordance with the recommendations in the Guide for the Care and Use of Laboratory Animals of the Guidelines for Animal Experimentation of Tohoku University, Sendai, Japan. The present study was approved by the Institution Animal Care and Use Committee of Tohoku University with a permitted No. 2011-noudou-5 and all efforts were made to minimize suffering [7]. Briefly, PPs or MLNs were cut into small fragments, which were gently pressed through a nylon mesh and washed three times in complete RPMI 1640 medium (Sigma, St. Louis, MO) supplemented with 10% FCS (Sigma). Residual erythrocytes were lysed by resuspending in hypotonic salt solution (0.2% NaCl). Next, harvested PP or MLN cells were subjected to hypertonic rescue in an equal volume of 1.5% NaCl. Finally, immune cells were fractionated using Lympholyte-mammal (Cedarlane, Hornby, Ontario, Canada) density gradient centrifugation, and the isolated immune cells were suspended in complete DMEM (Invitrogen, Tokyo, Japan) supplemented with 10% FCS (Sigma) and 50 µg/ml penicillin-streptomycin (Life Tech., Carlsbad, CA, USA).

### Mitogenicity Assay

The mitogenicity assay was performed as described [7]. Briefly, PPs or MLN immunocompetent cells were placed in a 96-well microplate (Costar, 2×10^5^ cells/well) and incubated at 5% CO_2_, 37°C for 48 h in complete RPMI 1640 medium (Sigma) supplemented with 2% FCS. Immune cells were stimulated with bifidobacteria or lactobacilli for 48 h. During the final hour of culture, the cells were radiolabeled with 9.25 kBq per well of [methyl-^3^H]-Thymidine (GE Healthcare, Tokyo, Japan). The cells were then harvested with a glass fibre filter (PerkinElmer Japan, Kanagawa, Japan). The [methyl-^3^H]-Thymidine incorporation was quantitated in a liquid scintillation counter (Beckman Instruments, Palo Alto, CA, USA). Results are presented as the SI, calculated with the following equation: [(counts per minute in treated cultures) − (counts per minute in background)]/[(counts per minute in control cultures) − (counts per minute in background)].

### Immunomodulatory Effect of Bacteria

Evaluation of the immunomodulatory activity of lactobacilli and bifidobacteria strains was performed using mononuclear cells from PPs and MLNs or PIE cells. In each case, cells were plated at a density of 1.5×10^6^ cells/well in 12-well type I collagen-coated plates (Iwaki, Tokyo, Japan). Bacteria were added to each well at a concentration of 5×10^8^ cells/ml. Cells were incubated with bacteria for 48 h. Expression of IL-6, IL-8, and MCP-1 was evaluated with RT-PCR as described below. In addition, expression levels of six TLR negative regulators, A20, Bcl-3, IRAK-M, MKP-1, SIGIRR, and Tollip, were evaluated in PIE cells. In some experiments, unlabeled anti-porcine TLR2-rabbit IgG (Santa Cruz Biotechnology, Santa Cruz, CA) was used in blocking experiments. Cultured porcine cells were incubated with unlabeled anti-TLR2 antibodies for 12 h before stimulation with bacteria.

### Anti-inflammatory Assay in PIE Cells

PIE cells were seeded at 3×10^4^ cells/12-well plate on type I collagen-coated plates (Iwaki) and cultured for 3 days. After changing medium, bacteria (5×10^7^ cells/ml) were added; 48 h later, each well was washed vigorously with medium at least three times to eliminate all stimulants, and then cells were stimulated with heat-stable ETEC PAMPs (equivalent to 5×10^7^ cells/ml) for 12 h.

### Quantitative Analysis of Expression with Real-time PCR

We performed two-step real-time quantitative PCR to characterize the expression of mRNAs in PIE cells and immune cells. Total RNA was isolated from each PIE or immune cell sample using TRIzol reagent (Invitrogen). All cDNAs were synthesized using a Quantitect reverse transcription (RT) kit (Qiagen, Tokyo, Japan) according to the manufacturer’s recommendations. Real-time quantitative PCR was carried out using a 7300 real-time PCR system (Applied Biosystems, Warrington, UK) and the Platinum SYBR green qPCR SuperMix uracil-DNA glycosylase with 6-carboxyl-X-rhodamine (Invitrogen). The primers for IL-6, IL-8, MCP-1, A20, SIGIRR, Tollip, Bcl-3, MKP-1, and IRAK-M used in this study were previously described [2], [9]. The PCR cycling conditions were 5 min at 50°C, followed by 5 min at 95°C, and then 40 cycles of 15 s at 95°C, 30 s at 60°C, and 30 s at 72°C. The reaction mixtures contained 2.5 µl sample cDNA and 7.5 µl master mix, which included the sense and antisense primers. Expression of β-actin in each sample was assessed, and the β-actin data were used as an internal control to normalize differences between samples and to calculate relative expression levels.

### ELISA

The concentrations of IL-6, IL-8, and MCP-1 secreted into the supernatant of PIE cell cultures 48 h after ETEC stimulation were determined using commercially available ELISA kits (porcine IL-6 ELISA kit [Ray-Bio, Norcross, GA], IL-8 immunoassay kit [Biosource, Camarillo, CA], and porcine MCP-1 ELISA kit [E101–800, Bethyl Laboratories, Inc. Montgomery, TX, USA]), according to the manufacturers’ instructions.

### Western Blotting

PIE cells (1.8×10^5^ cells/dish) cultured in 60-mm dishes were stimulated with bacteria as described previously. PIE cells were then washed and stimulated with ETEC for the indicated times, washed three times with PBS, and resuspended in 200 µl CelLytic M cell lysis reagent (Sigma) including protease and phosphate inhibitors (Complete Mini, PhosSTOP; Roche, Mannheim, Germany). Cells were transferred into Eppendorf tubes and boiled for 5 min at 95°C. Protein concentration was measured using the bicinchoninic acid protein assay kit (Pierce, Rockford, IL). Total protein samples (2 µg/sample) were loaded onto 10% SDS-polyacrylamide gels. Separated proteins were electrophoretically transferred to a nitrocellulose membrane. Phosphorylation of p38 and JNK and ERK MAPKs, and IκBα degradation were evaluated using anti-phosphorylated p38, anti-phosphorylated JNK, anti-phosphorylated ERK, and anti-IκB antibodies, respectively (Cell Signaling Technology, Beverly, MA), according to the manufacturer’s instructions. The optical density of each band was measured using Image J (National Institutes of Health, Bethesda, MD).

### Statistical Analysis

Statistical analyses were performed using the GLM and REG procedures available in the SAS computer program (SAS, 1994). Comparisons between mean values were carried out using one-way analysis of variance and Fisher’s least-significant-difference test. For these analyses, P values of <0.05 were considered significant.
